# Effects of Low-Fat Diets Differing in Protein and Carbohydrate Content on Cardiometabolic Risk Factors during Weight Loss and Weight Maintenance in Obese Adults with Type 2 Diabetes

**DOI:** 10.3390/nu8050289

**Published:** 2016-05-12

**Authors:** Nerylee Watson, Kathryn Dyer, Jonathan Buckley, Grant Brinkworth, Alison Coates, Gaynor Parfitt, Peter Howe, Manny Noakes, Karen Murphy

**Affiliations:** 1 Alliance for Research in Exercise, Nutrition and Activity, Sansom Institute for Health Research, University of South Australia, GPO Box 2471, Adelaide 5001, SA, Australia; nerylee.watson@mymail.unisa.edu.au (N.W.); Kate.Dyer@unisa.edu.au (K.D.); Jon.Buckley@unisa.edu.au (J.B.); Alison.Coates@unisa.edu.au (A.C.); Gaynor.Parfitt@unisa.edu.au (G.P.); 2 Food and Nutrition, Commonwealth Scientific and Industrial Research Organization, PO Box 10041, Adelaide 5000, SA, Australia; grant.brinkworth@csiro.au (G.B.); manny.noakes@csiro.au (M.N.); 3 Clinical Nutrition Research Centre, School of Biomedical Sciences and Pharmacy, University of Newcastle, University Drive, Callaghan 2308, NSW, Australia; Peter.Howe@newcastle.edu.au

**Keywords:** type 2 diabetes, glycaemic, cardiovascular disease risk, weight loss, weight maintenance, obesity

## Abstract

Despite evidence for the benefits of higher-protein (HP) diets in weight loss, their role in type 2 diabetes mellitus (T2DM) management and weight maintenance is not clear. This randomised study compared the effects of a HP diet (38% carbohydrate, 30% protein, 29% fat) to a isocaloric higher-carbohydrate diet (HC: 53%:21%:23%) on cardiometabolic risk factors for 12 weeks in energy restriction (~30% reduction) followed by 12 weeks of energy balance whilst performing regular exercise. Outcomes were measured at baseline and the end of each phase. Sixty-one overweight/obese adults (BMI (body mass index) 34.3 ± 5.1 kg/m^2^, aged 55 ± 8 years) with T2DM who commenced the study were included in the intention-to-treat analysis including the 17 participants (HP *n* = 9, HC *n* = 8) who withdrew. Following weight loss (M ± SEM: −7.8 ± 0.6 kg), there were significant reductions in HbA1c (−1.4% ± 0.1%, *p* < 0.001) and several cardiometabolic health risk factors. Improvements were sustained for 12 weeks when weight was stabilised and weight loss maintained. Both the HP and HC dietary patterns with concurrent exercise may be effective strategies for weight loss and weight maintenance in T2DM although further studies are needed to determine the longer term effects of weight maintenance.

## 1. Introduction

The management of type 2 diabetes mellitus (T2DM) focuses on achieving and maintaining a healthy weight and increasing physical activity to obtain better glycaemic control and reduce cardiovascular risk factors. Weight loss of 5%–10% of initial weight from calorie restriction, independent of dietary pattern, has been shown to improve cardiometabolic health [1] and HbA1c [2] in overweight and obese individuals including those with T2DM. Conjecture remains regarding the dietary pattern which optimizes weight loss and metabolic benefits in T2DM, however current evidence suggests that low-fat, higher-protein (HP) diets, where some dietary carbohydrate is substituted with protein, are equally beneficial to diets with higher-carbohydrate (HC) content, at least in energy restriction [3,4,5]. Most studies comparing a HP diet with a HC diet focus on weight loss only and there is limited support for specific dietary patterns in energy-balance, after successful weight stabilization, when weight loss is no longer the objective. Furthermore there is a lack of evidence investigating whether the benefits accrued in weight loss are maintained during energy-balance, particularly when diet is combined with exercise, as is recommended for people with T2DM, where the exercise might continue to provide improvements in cardiometabolic risk factors during weight maintenance. Overall, there is limited literature examining the effects of HP dietary patterns in energy-balance on glycaemic control and cardiometabolic health in people with T2DM, particularly in the context of concurrent exercise.

The aim of this study was therefore to compare the effects of isocaloric HP and HC diets, combined with regular moderate intensity exercise, on glycaemic control and cardiometabolic risk factors in overweight and obese adults with T2DM initially following 12 weeks of active weight loss, and then to re-evaluate the outcomes following 12 weeks of energy balance without the influence of weight change. By including an exercise component, we are able to determine the effects of the dietary patterns when administrated as part of a holistic lifestyle intervention as recommended in the management of T2DM.

## 2. Materials and Methods

### 2.1. Participants, Randomization, and Study Design

The full protocol including eligibility criteria, study design, and a detailed description of the outcome measures has been reported previously [6]. Briefly, 61 overweight/obese adults (BMI (body mass index) ≥ 25 kg/m^2^; aged 18–70 years) with T2DM (HbA1c 6.5%–10.5%) commenced a 24 weeks; two-arm parallel-group lifestyle intervention study. Prior to commencement, participants obtained approval from their general practitioner to participate in the study before their written informed consent was obtained. The study was approved by the University of South Australia Human Research Ethics Committee (Approval no. #30653) and the CSIRO Human Research Ethics Committee (HREC: 12/18) and registered with the Australian New Zealand Clinical Trials Registry (#12613000008729).

Participants were randomly allocated to the consumption of either a HP diet (*n* = 32) or a HC diet (*n* = 29) by the process of minimization [7] stratified by age, gender, and BMI. There were two phases: a 12 weeks hypocaloric, weight loss phase (phase 1), immediately followed by a 12 weeks eucaloric, weight maintenance phase (phase 2). Participants attended clinic appointments at baseline and the end of each study phase (Weeks 0, 12, and 24) for outcome assessments. The primary outcome was glycaemic control (% HbA1c) and secondary outcomes were BMI, body composition (measured by dual energy X-ray absorptiometry (DEXA)), waist circumference, systolic blood pressure (SBP), diastolic blood pressure (DBP), fasting blood biochemistry (lipids, glucose, insulin and C-reactive protein (CRP)), insulin resistance (calculated using the Homeostasis Model Assessment 2 (HOMA2-IR)) [8], and changes in diabetes medication usage quantified by a medication effect score (MES) [9]. Isolated CRP values >10 mg/L were excluded from analysis as these values may represent an acute infection or inflammation [10].

### 2.2. Diet and Exercise Interventions

The diets were isocaloric with the planned macronutrient content for the HP diet being 32% of total energy as protein, 33% carbohydrate, and 30% total fat (<10% as saturated fat) and the HC diet was 22% protein 51% carbohydrate, and 22% total fat (<10% as saturated fat). Using the Schofield Equation, based on sex, age, and initial body weight [11], individual estimated energy requirements (EER) were calculated and a moderate energy restriction of ~30% of the EER was prescribed (equating to ~6000–7000 kJ/day) to facilitate weight loss for phase 1 (Weeks 0 to 12). To achieve weight maintenance for phase 2 (Weeks 12 to 24), energy intake was increased whilst retaining the prescribed macronutrient profile. Participants received comprehensive dietary advice from a qualified dietitian at baseline and every two weeks during the study and were provided with core study foods corresponding to their assigned dietary pattern: fresh lean pork, breakfast cereal, mixed grain bread, fat-reduced cheese (HP only), and raw almonds (HP only). Daily semi-quantitative food records were completed to guide dietary intake and to permit subsequent dietary analysis. Analysis was based on seven consecutive days from every two-weekly food record and the mean macronutrient intake for the weight loss and the weight maintenance phases was reported. Analysis was performed using the computerized database (FoodWorks^®^ Professional Edition, version 7, 2012, Xyris Software, Highgate Hill, Australia).

Throughout the study, participants were asked to undertake a minimum of 30 min of moderate intensity aerobic exercise, five times per week (150 min/week). To guide participants, moderate intensity was defined as corresponding to a rating of perceived exertion of 13 (*i.e.*, somewhat hard) on Borg’s 6–20 rating of perceived exertion scale [12]. Participants completed physical activity logs to monitor compliance. The values were summed to give the total minutes of exercise per phase for each participant. Participants also undertook a treadmill exercise at each clinic visit to allow for assessment of changes in physical fitness. The treadmill exercise test consisted of a three-minute walk/run at a 0° gradient with the speed, which was hidden from view, adjusted until the participant perceived the intensity as RPE-13. The speed was recorded to allow for assessment of changes in cardiorespiratory fitness, with increases in walk/run speed at RPE-13 being indicative of improvements in cardiorespiratory fitness. The treadmill speed at which RPE13 was reached was recorded at each clinic visit.

### 2.3. Statistical Analysis

Statistical analyses were performed using SPSS version 21.0 (SPSS Inc., Chicago, IL, USA). Non-normally distributed data (HbA1c, weight, waist, BMI, fat mass, glucose, insulin, HOMA2-IR, SBP, DBP, CRP, triglycerides, and HDL (high-density lipoprotein)) were log transformed before analysis. Dietary data, physical activity logs and baseline characteristics between the groups were compared using independent student *t*-tests for continuous variables and chi-square tests for categorical variables. Where there was only one or two weeks of missing physical activity data per phase, mean substitution for that phase was used. The effects of the different interventions over time were assessed using an intention-to-treat analysis (including all participants who commenced the study) using restricted maximum-likelihood, linear mixed effects modelling with an unstructured covariance. Treatment was the between-subject factor and time was the repeated within-subject measurement. Where there was a significant main effect, post-hoc comparisons were performed with Bonferroni’s adjustments for multiple comparisons to determine differences between group means. Dietary, physical activity, and baseline data are presented as mean ± SD with all other variables reported as mean ± SEM. Statistical significance was set at *p* < 0.05.

## 3. Results

### 3.1. Participants

Of the 63 randomised participants, two withdrew prior to baseline assessments and were not included in the analysis, leaving 61 participants who commenced the study (HP: *n* = 32, HC: *n* = 29). A total of 17 participants withdrew (HP: *n* = 9, HC: *n* = 8) over the course of the study, but were included in the mixed-model analysis. Reasons for withdrawing were: unable to comply with the intervention (HP: *n* = 3, HC: *n* = 2); work commitments (HP: *n* = 2, HC: *n* = 2); illness/death in the family (HP: *n* = 3, HC: *n* = 1); personal reasons (HC: *n* = 2); no reason given (HP: *n* = 1), and loss to follow-up (HC: *n* = 1). At baseline, compared to completers, non-completers had a greater total fat mass (44.7 ± 10.6 kg *vs.* 36.9 ± 9.9 kg, *p* = 0.01) and lower diastolic blood pressure (75 ± 7 mmHg *vs.* 80 ± 8 mmHg, *p* = 0.02). Although non-completers also had a higher mean BMI (36.5 ± 5.2 kg/m^2^ *vs.* 33.5 ± 4.8 kg/m^2^, *p* = 0.04) their weight was not significantly different to completers (102.2 ± 16.1 kg *vs.* 98.2 ± 17.2 kg, *p* = 0.40). Sensitivity analysis was conducted for the BMI, fat mass (kg), and DBP variables using two methods: all participants who commenced the study and completers only. Both analyses showed the same pattern of outcomes with a time effect (*p* < 0.001) but no differences between the groups (*p* ≥ 0.51) or group by time interactions (*p* ≥ 0.07). This implies that our linear mixed effects analysis has accurately modelled the missing data and the baseline differences have not had an adverse influence on our conclusions. There were no differences in baseline characteristics between the diets (Table 1).

### 3.2. Dietary Analysis and Physical Activity Compliance

Based on the dietary data collected, participants achieve good compliance to their allocated dietary prescription (Table 2). Total energy intake was similar between the groups for both intervention phases. Compared to the HC group, the HP group consumed significantly more protein and less carbohydrate and fiber (*p* < 0.05 for each nutrient) for each phase. As expected, the total fat intake in the HP diet was higher than the HC diet as a result of larger meat portions and the inclusion of reduced-fat cheese and almonds, however both groups met the dietary recommendations for saturated fat intake (≤10% of energy intake) [13].

Physical activity compliance indicated that both groups exceeded their requirements of 150 min/week during both phases with no significant difference between groups (*p* > 0.08 for each phase, Table 2). Compared with baseline, treadmill speed at RPE 13 had increased significantly following phase 1 (HP: 0.67 ± 0.12 km/h; HC: 0.53 ± 0.13 km/h; *p* < 0.001 (time)) and phase 2 (HP: 0.35 ± 0.12 km/h; HC: 0.28 ± 0.13 km/h; *p* = 0.003 (time)), with no difference between groups (*p* = 0.70 group × time interaction, Table 3). As the change in treadmill speed typifies increase in fitness, the linear mixed effects analysis was repeated with change in treadmill speed included as a covariate to partially explain any independent effects from exercise. Any variable which has shown additional benefits has been reported.

### 3.3. Body Weight

During phase 1, both the HP and HC hypocaloric diets achieved significant reduction in weight (*p* < 0.001) of −8.0 ± 0.8 kg and −7.6 ± 0.8 kg, respectively (Table 3). The proportion of participants who lost ≥5% and ≥10% of initial body weight was similar between the diets (≥5%: HP 19 of 23, 83%; HC 18 of 22, 82%; *p* > 0.99, ≥10%: HP 8 of 23, 35%; HC 4 of 22, 18%; *p* = 0.21). Weight loss was maintained in phase 2 (*p* = 0.36) with no significant difference between the groups for either phase (*p* = 0.47 for group × time interaction, Table 3).

### 3.4. Glycaemic Control

Following phase 1, HbA1c (%) decreased in both groups (*p* < 0.001) and remained stable for phase 2 (*p* > 0.99) with no difference between the groups at either time points (*p* = 0.19 group × time interaction, Table 3). MES decreased over time (*p* = 0.02), with no significant difference between the groups (*p* = 0.43) or group × time interaction (*p* = 0.08, Table 3). Fasting blood glucose and insulin levels decreased in both groups following phase 1 (*p* < 0.001 for both variables), with no change following phase 2 (*p* ≥ 0.81), nor any difference between the groups at either time points (*p* ≥ 0.52 group × time interactions, Table 3).

A sub-group analysis examined the diet differences for those participants who did not change their diabetes medication (*i.e.*, medication effect score did not change) at any time point in the study (*n* = 45, HP *n* = 23, HC *n* = 22). Those who withdrew from the study were considered not to have changed their medication. HbA1c indicated no overall difference between the groups (*p* = 0.13) but a time effect (*p* < 0.001) and a group by time interaction (*p* = 0.03) was found. *Post hoc* analysis (with Bonferroni’s adjustment for multiple comparisons) showed that while both groups significantly reduced HbA1c following weight loss (HP: −1.64% ± 0.21%, HC: −0.96% ± 0.20%, *p* < 0.001 (time)), the decrease in the HP group was greater than the HC group (mean difference: −0.74% ± 0.29%, *p* = 0.02) at this time point. This significant group by time interaction was not seen in the primary analysis, which included those with medication changes, thereby suggesting that changes in medication influenced the results. Sub-group analysis showed a similar pattern to the primary analysis (*i.e.*, including those with medication changes) with a time effect (*p* < 0.001) but no group (*p* ≥ 0.37) or group by time interactions (*p* ≥ 0.20) for glucose, insulin, and weight. When the overall change in treadmill speed was added as a covariate, all variables significantly decreased over time (*p* < 0.001) but there were no significant differences seen for groups (*p* ≥ 0.18), group by time interactions (*p* ≥ 0.11), or group by time by change in treadmill speed interactions (*p* ≥ 0.35).

### 3.5. Body Composition

Waist circumference and fat mass significantly decreased with weight loss (*p* < 0.001) and remained stable during phase 2 (*p* ≥ 0.16) with no differences between the groups for any time points (*p* ≥ 0.47 for group × time interaction, Table 3). There was a significant reduction in lean body mass during phase 1 (*p* < 0.001), with no further significant reductions in lean mass following phase 2 (*p* > 0.99) with both groups showing a similar response (*p* = 0.53 group × time interaction, Table 3).

### 3.6. Blood Pressure (BP), Insulin Resistance, and CRP

Systolic (SBP) and diastolic (DBP) blood pressure fell following phase 1 (*p* < 0.001) and remained stable for phase 2 (*p* ≥ 0.77) with no difference between groups for either variable (*p* ≥ 0.07, Table 3). Antihypertensive medication dosage was reduced for seven participants (HP *n* = 5, HC *n* = 2) and increased for one participant in the HC group by their health professional. A sub-group analysis was conducted for those participants who did not change their blood pressure medication at any time point in the study with those who withdrew from the study considered not to have changed their medication (*n* = 53: HP *n* = 27, HC *n* = 26). Results for SBP were similar to the primary analysis with no significant group (*p* ≥ 0.28) or group by time interaction (*p* = 0.32) suggesting that change in BP medication did not have an effect on the results for SBP. However, there was a group by time interaction for DBP (*p* = 0.02) indicating a higher overall reduction in the HP group (−9.1 ± 1.3 mmHg) than the HC group (–4.0 ± 1.3 mmHg) with the difference (−5.0 ± 2.3 mmHg) being significant (*p* = 0.04). However, this significance was lost when the change in treadmill speed was added as a covariate: group by time interaction (*p* = 0.38); group by time by change in treadmill speed interaction (*p* = 0.54).

HOMA2-IR decreased in phase 1 (*p* < 0.001) and remained stable during phase 2 (*p* > 0.99) with no difference between the groups during either phase (*p* = 0.45, Table 3). CRP significantly reduced following phase 1 (*p* = 0.03) and remained stable at the end of phase 2 (*p* = 0.16) with no difference between the groups (*p* = 0.99 group × time interaction, Table 3). Changes in CRP positively correlated with changes in HbA1c (*r* = 0.52, *p* = 0.001) which remained significant after adjusting for age, years with diabetes, and baseline weight.

### 3.7. Blood Lipids

There was a significant diet by time interaction for total cholesterol (*p* = 0.049); with the HP diet, it significantly decreased in phase 1 (*p* < 0.001) and, although it significantly rebounded during phase 2 (*p* = 0.04), there was an overall decrease from baseline to the end of phase 2 (*p* = 0.01, Table 4). There were no changes in total cholesterol for the HC diet (*p* ≥ 0.20). LDL (low-density lipoprotein) decreased following phase 1 (*p* = 0.02) but, over the course of the study, there was no change from baseline values (*p* = 0.21) and no difference between the diets (*p* = 0.18 diet × time interaction, Table 4). There was a small significant increase in HDL during phase 2 (*p* = 0.01) but it was not significantly different between diets (*p* = 0.14, Table 4). Triglycerides decreased following phase 1 (*p* < 0.001) and remained stable during phase 2 (*p* = 0.90) with no differences between the diets (*p* = 0.15 diet × time interaction, Table 4).

Lipid-lowering medication was reduced in four participants (HP *n* = 1, HC *n* = 3) and increased in one participant in the HP group. A sub-group analysis examining dietary differences in lipids for those participants who did not change their cholesterol lowering medication at any time point in the study (*n* = 57: HP *n* = 31, HC *n* = 26) was conducted, with those who withdrew from the study considered not to have changed their medication. Results for total cholesterol (TC) did not show a group effect (*p* = 0.81) but a time (*p* < 0.001) and group by time interaction (*p* = 0.04) as with the primary analysis. For TC, when change in treadmill speed was added as a covariate, the group by time interaction was no longer significant (*p* = 0.60) and there was no group by time by change in treadmill speed interaction (*p* = 0.34). Changes in medication did not influence results for HDL, LDL, and triglycerides.

## 4. Discussion

In this study both HP and HC energy-restricted, isocaloric diets combined with regular moderate intensity exercise provided substantial improvements in weight, glycaemic control, and cardiometabolic risk factors in overweight and obese adults with T2DM. Furthermore, these improvements were maintained during a subsequent 12 weeks period when weight remained stable, allowing for two distinct phases to compare outcomes.

Improvements in glycaemic control were the primary outcome of this study. Our primary analysis showed substantial reductions in HbA1c, fasting blood glucose levels, and fasting insulin with no differences between the groups which is consistent with similar weight loss studies [3,4] even when adjustments for changes in medication have been made [5]. Another study reported that additional benefits for a lower-carbohydrate diet were only seen in participants whose baseline mean HbA1c levels were higher (>7.8%) even though the reduction in MES in that group was two-fold greater [14]. Diabetes medication is often titrated in response to fluctuations in glycaemic control. The change in medication observed in our participants was small, as indicated by the MES, but may have obscured any benefits for a HP diet. After conducting a sub-group analysis, including only those who did not change their diabetes medication, we found that the HP group achieved a 0.7% greater reduction in HbA1c than the HC group following phase 1 with no difference between the groups for weight loss. Interestingly, this supports a previous study where a 0.7% greater reduction in HbA1c was found after following a hypocaloric lower-carbohydrate diet (14%Carbohydrate:27%Protein:54%Fat) compared to an isocaloric diet (50%C:19%P:25%F) for 24 weeks [14]. In contrast to that study, our dietary intervention consisted of a moderate carbohydrate difference between the groups of 15% of total energy (or approximately 50 g carbohydrate) suggesting that even modest carbohydrate restriction can elicit glycaemic improvements. Following weight maintenance, the difference between the means (0.6%) in the sub-group analysis did not reach statistical significance suggesting we were under-powered to detect this level of change. However, this result is of clinical significance and is consistent with a five weeks cross-over study that reported their HP diet (30%P:40%C:30%F) achieved a 0.5% greater reduction in HbA1c than the HC (15%P:55%C:30%F) where participants were not treated with diabetes medication and were weight stable [15]. The benefits we found in our sub-group analysis demonstrate the need for caution when interpreting results in T2DM studies where diabetes medication dosage fluctuates. Nevertheless, including participants with or without medications in studies is necessary to generalize the results.

While most studies focus on weight loss there is currently limited evidence available to support prescriptive higher-protein diets with higher-carbohydrate diets in adults with T2DM which incorporate a concurrent exercise protocol and report outcomes following successful weight stabilization over an appropriate duration to see changes. Our study was designed to promote weight loss initially and then re-evaluate the outcomes following 12 weeks of energy-balance without the influence of weight change so as to better isolate the effects of diet composition independent of any effects of weight loss. The 12 weeks duration was considered sufficient time to allow for any changes in HbA1c to be evident. The present study showed that there was no significant change in HbA1c when energy intake was increased for weight maintenance while weight was maintained for 12 weeks. Both dietary patterns increased carbohydrate similarly intake by 17–20 g. This is in contrast to findings of a previous study where HbA1c rebounded after a nine month weight maintenance phase [5]. Although this previous study had a longer weight maintenance period, we achieved a 2.5-fold greater weight loss prior to the weight maintenance period (−8 kg *vs.* −3 kg) resulting in a greater reduction in HbA1c (−1.5% *vs.* −0.5%) which provided us with two very distinct phases with which to compare results. The sustained improvement in HbA1 reported in our study (−1.4%) is of clinical significance as it has been reported that the risk of a stroke is reduced by 12%, myocardial infarction by 14%, and diabetes-related deaths by 21% for each 1% sustained decrease in HbA1c [16]. The magnitude of HbA1c reduction we observed is similar to the magnitude of reduction that is expected using oral antihyperglycaemic medication in monotherapy [17]. Apart from the obvious financial benefits, glycaemic control through lifestyle modifications has many advantages over pharmacological usage. Side effects associated with antihyperglycaemic medication, including gastrointestinal upsets, hypoglycemia, renal impairment, and weight gain, can be reduced or eliminated as medication is lowered or stopped. Coupled with the improvement for HbA1c there was a significant reduction in antihyperglycaemic medication use over the course of the study. This was a smaller reduction than seen in other studies which have utilized the same MES method that have reported greater reductions (−0.5 to −1.24), but these studies have used diets with substantially lower-carbohydrate intakes (14%–20% carbohydrate) [9,18].

There were no differences between the groups for changes in body composition: total fat mass (FM) and fat free mass (FFM) accounted for 79% and 20% of weight loss respectively. This is consistent with a previous 16 weeks study of adults with T2DM who followed a HP or HC diet with or without resistance training (RT) which showed that FM loss accounted for 80% of weight lost in the RT groups and 77% for the diet only groups and FFM loss accounted for 20% of weight loss in the RT groups and 23% for the diet only groups [3]. Another 20-week study of overweight and obese women with polycystic ovary syndrome, which displays similar cardiometabolic pathology as T2DM (abdominal obesity, insulin resistance, hypertension, and dyslipidemia), also concluded that the inclusion of exercise to a hypocaloric diet provided greater FM loss and preservation of some FFM [19]. Our covariate analysis did not identify any additional benefits in body composition from exercise. This may be due to our shorter weight loss phase of 12 weeks whereas the above mentioned studies were of 16–20 weeks duration. Furthermore, with both groups achieving similar weight loss and improvements in fitness, as evidenced from improved treadmill speed at the same RPE, it may be possible that the moderate weight loss of 8 kg may have masked any independent effect that exercise may have had. Importantly, we showed that body composition benefits remained when weight lost was sustained.

Apart from weight loss and adequate glycaemic control, improving BP, insulin resistance, and CRP and lipid profiles can also play an important role in lowering CVD risk in diabetes. Mean BP values at baseline indicate that our participants were within the recommendations set out in the American Diabetes Association’s (ADA) guidelines for T2DM management [20]. Nevertheless, BP improved following weight loss with both diets which is consistent with previous findings [3]. BP medication was adjusted during the study and a sub-group analysis for those who did not change their medication revealed the HP group reduced DBP to a greater degree than the HC group. Notably, the significance was lost when adjusted for the change in treadmill speed suggesting that exercise may have provided additional benefits beyond the weight loss or dietary patterns for those who did not change their blood pressure medication. Moreover, BP remained stable during phase 2. A previous study showed that BP improvements following a hypocaloric weight loss diet did not rebound in a weight stabilization phase when energy intake was increased in overweight and obese adults without T2DM [21]. The energy-balance phase of that study was short (4 weeks) so our study extends this evidence to include a 12 weeks weight maintenance phase and individuals with T2DM. The overall reduction in SBP (−11 mmHg) is clinically noteworthy as a 10 mmHg decrease is associated with a 11% reduction in risk for myocardial infarction and 15% reduction in risk for diabetes-related deaths with benefits also seen in normotensive individuals [22].

One of the independent predictors of CVD is insulin resistance through its association with hyperglycemia, hyperinsulinemia, hypertension, and dyslipidemia [23]. Using the HOMA2-IR cut-off value for insulin resistance of >1.8, as determined by the BRAMS study [24], our participants were substantially insulin resistant at baseline. However, following weight loss in phase 1, HOMA2-IR indices had markedly decreased which supports previous findings [25]. It has been reported that the risk of CVD increases by 56% with a 1 unit increase in HOMA-IR [26], therefore the overall reduction of −1.44 and −1.04 for the HP and HC diets respectively is considered clinically important. Following phase 2 in our study, HOMA2-IR scores indicated that insulin resistance had normalized (<1.8 for both groups), denoting a reduction in CVD risk.

CRP, a major acute-phase protein associated with chronic systemic inflammation, has been associated with obesity (particularly abdominal adiposity) and insulin resistance and may predict coronary heart disease risk [27]. In our study, CRP significantly decreased following weight loss, thereby supporting the current evidence [28] of there not being any difference between the HP or HC diets, which is in agreement with other studies regarding T2DM [3,18]. Increases in CRP are also related to worsening glycaemic control in T2DM [29] and we report a strong positive correlation between reductions in CRP levels and reductions in HbA1c. Improvements in CRP remained during phase 2.

After an initial reduction in phase 1, total cholesterol and LDL levels rebounded in phase 2 when weight was stabilised, whereas HDL improved during phase 2. Interestingly, the pattern of change observed is similar to studies previously reported in T2DM individuals [4,5] and overweight and obese adults [30] that show lipids decline with caloric restriction but rebound with weight stabilization and energy balance. At baseline, triglycerides level were above current ADA recommendations, but decreased in phase 1 to within recommended levels with no difference between the diets. This result is consistent with the findings from a meta-analysis of T2DM studies [31] but not from a meta-analysis of overweight and obese adults where HP diets resulted in a significant 0.23 mmol/L greater reduction in triglyceride levels than standard protein diets [32]. A previous study reported that a carbohydrate restricted diet provided greater reductions in triglycerides than a calorie restricted diet (−55% *vs.* −28%) despite a similar 4.3% weight loss [33], which is supported in other studies where carbohydrate content was more severe [18]. Triglyceride levels remained stable during phase 2. It was interesting to note the additional benefits exercise appeared to exhibit on HDL results. This supports the findings from a meta-analysis of 35 studies which reported that regular aerobic exercise (for at least 8 weeks) modestly increased HDL by 2.53 mg/dL (95%CI 1.36 to 3.70) (equates to 0.065 mmol/L) more than non-exercising participants, but exercise duration needed to be ≥120 min/week [34]. However, this is contrary to another meta-analysis of 17 studies which found no difference between HDL between the aerobic exercise groups and non-exercising groups [35].

Comparable weight loss and the participants’ good compliance to their allocated macronutrient profile, energy intakes, and physical activity adds strength to our study as we can attribute metabolic changes to the differences between the intervention groups. The effective stabilization of weight in our 12-week maintenance phase is another strength of this study as it enabled further evaluation of outcomes without the confounders of energy imbalances and weight regain. Dietary compliance can be ascribed to the intensive dietetic and professional support given throughout the study, as evidence suggests that achieving weight goals is more successful when instructions are led by a registered dietitian [36]. We provided two-weekly individual visits, prescriptive diet plans, recipe books, regular monitoring of weight, and core study foods. However, it may limit the potential translation of results from the study into clinical practice where such intensive dietetic support may not be realistic. To ensure our results would be of clinical interest to health professionals, our dietary patterns were designed to incorporate all core food groups with the HP diet including a moderate carbohydrate intake and low to medium glycaemic index carbohydrates.

The lack of difference seen between the diets may have been partly due to the macronutrient composition. Circulating blood glucose as a product of carbohydrate metabolism could have had an impact on HbA1c, glucose, insulin, and triglyceride levels and our diets were not planned to have the differences in carbohydrate intake which are often seen in studies which report greater benefits for a HP diet over a HC diet [14]. However, it is likely that weight loss from the lifestyle intervention is the driving force for the metabolic changes as it was in this phase that most variables demonstrated significant changes. A 2%–10% reduction in body weight is associated with reductions in HbA1c, BP, and triglycerides and increased HDL cholesterol [2]. In this study both diets achieved a moderate weight loss of ~8% and met the exercise protocol which has been reflected in the results for these outcomes.

Limitations in this study should be considered when interpreting the results. Firstly, our sample size may not have provided the statistical power to detect smaller changes between groups, particularly for sub-group analysis. Secondly, the differences in the outcomes cannot be entirely attributed to differences in the protein/carbohydrate ratio between the two groups and other dietary factors other than carbohydrate and protein could have also influenced the results. The total fat and saturated fat (SFA) intakes in the HP group were 7%–8% and 3% higher, respectively. This was a result of larger meat portions and the inclusion of reduced-fat dairy and almonds in the HP group. Fibre intake was higher in the HC group (~3.3 g/day). However, we are comparing two dietary patterns with concurrent exercise rather than specific nutrients. Although our diets were prescriptive, participants had choices within some types of food (e.g., non-starchy vegetables, fruit). It would be impossible to assign changes in outcomes to a specific nutrient when food is a combination of nutrients, all of which may impact health outcomes. Current Australian dietary guidelines recommend total fat intake to account for 20%–35% of energy, SFA < 10% of energy intake, and an adequate intake of fibre being 25–30 g/day [37]. Therefore our dietary patterns are within the guidelines, although SFA was ~10% during weight maintenance. The POUNDS LOST trial of overweight /obese adults (*n* = 424) compared four isocaloric, energy-restricted diets: two were low fat (26% en (energy)–28% en) with either low protein (18% en–20% en) or high protein (21% en–23% en) and two were high fat (33% en–35% en) with either low protein (18% en–20% en) or high protein (21% en–23% en) and saturated fat intake was 7% en–11% en [38]. Their results led to the conclusion that the significant decreases in fat mass and abdominal fat were dependent on energy intake rather than macronutrient distribution as there were no dietary differences. Another study of obese adults with T2DM (*n* = 115) following hypocaloric diets that were either low-carbohydrate (14%C:27%P:54%F:10%SFA) or a HC (50%C:19%P:25%F:7.5%SFA) over 24 weeks found that both diets improved body composition, BP, HOMA2-IR, and CRP with the LC diet exhibiting greater improvements in glycaemic control, triglycerides, and HDL [14]. Thirdly, with both groups completing the same exercise protocol, it preludes the ability to separate the effects of diet and physical activity on the outcomes examined. Our aim was to compare the effects of the two dietary patterns when administered as part of a holistic lifestyle modification program with both diet and exercise being the key elements to the management of T2DM.

## 5. Conclusions

In summary, both energy-restricted dietary patterns combined with moderate intensity exercise, as a holistic lifestyle modification, achieved similar weight loss, improvements in glycaemic control, and reductions in key cardiometabolic risk factors. Furthermore, we found that the HP diet provided greater reductions in HbA1c when diabetes medication was unchanged, and we recommend that this should be considered in future studies. Moreover, we have shown that these improvements remained in energy-balance when weight loss and exercise was sustained for 12 weeks. With CVD responsible for nearly 70% of diabetes-related deaths [39], our results are noteworthy. Overall, these data support current evidence that lifestyle modifications combining diet and exercise are key strategies for the management of T2DM with both dietary patterns, outlined in this study, equally as effective in reducing CVD risk. Further studies are needed to determine the longer term effects of weight maintenance and to integrate the lifestyle program components of this study within cost-effective community-based delivery models.

## Figures and Tables

**Table 1:** Baseline characteristics for the study participants.

	Higher-Protein Diet *N* = 32	Higher-Carbohydrate Diet *N* = 29
Age (years)	54 ± 8	55 ± 8
Sex—Male (%)	17 (53)	16 (55)
Sex—Female (%)	15 (47)	13 (45)
Years with T2DM	7.9 ± 6.0	6.5 ± 4.2
Body Composition		
Body Weight (kg)	97.3 ± 17.1	101.5 ± 16.6
BMI (kg/m^2^)	34.3 ± 5.4	34.4 ± 4.7
Waist Circumference (cm)	112.8 ± 12.6	112.5 ± 12.3
Total Fat Mass (kg) *	38.5 ± 10.4	39.9 ± 11.0
Total Lean Mass (kg) *	58.8 ± 12.4	60.2 ± 11.7
Glycaemic Control		
HbA1c (%)	8.0 ± 1.3	8.1 ± 1.5
Fasting Glucose (mmol/L)	9.1 ± 3.2	9.3 ± 3.5
Cardiometabolic Health Risk Factors		
Systolic Blood Pressure (mmHg)	131.8 ± 13.2	135.1 ± 8.3
Diastolic Blood Pressure (mmHg)	78.4 ± 8.2	79.0 ± 7.1
Fasting Insulin (mU/L) ^^^	21.7 ± 14.8	22.8 ± 12.6
HOMA2-IR ^§^	2.9 ± 1.6	2.8 ± 1.4
Total Cholesterol (mmol/L)	4.7 ± 0.9	4.5 ± 0.8
LDL (mmol/L) ^¥^	2.7 ± 0.9	2.5 ± 0.6
HDL (mmol/L)	1.2 ± 0.3	1.2 ± 0.3
Triglycerides (mmol/L)	2.0 ± 1.2	2.0 ± 1.0
C-Reactive Protein (mg/L) ^†^	3.7 ± 3.9	4.6 ± 3.8
Medications		
Medication Effect Score	1.11 ± 0.99	1.22 ± 0.99
Metformin *n* (%)	18 (58)	18 (64)
Sulphonylureas *n* (%)	5 (16)	5 (18)
GLP-1 Agonists *n* (%)	2 (7)	2 (7)
DPP-4 Inhibitors *n* (%)	2 (7)	3 (11)
Combination ^#^ *n* (%)	3 (10)	1 (4)
Insulin *n* (%)	6 (19)	6 (21)
Lipid Lowering Medication *n* (%)	16 (52)	18 (64)
Antihypertensive Medication *n* (%)	19 (61)	12 (43)
Physical Activity		
Treadmill Speed (km/h)	4.6 ± 1.1	4.7 ± 0.7

Data represents mean ± SD. All *p* values are >0.05 for between group differences based on independent *t*-tests. *: Dual-energy X-ray absorptiometry scans were not performed on one participant (HC *n* = 28); ^^^: Fasting insulin was excluded for one participant (HP *n* = 31); ^§^: HOMA2-IR was unable to be calculated for 12 participants on insulin therapy and one participant due to high baseline fasting insulin level (HP *n* = 25, HC *n* = 23); ^¥^: LDL-C not calculated for five participants (HP *n* = 29, HC *n* = 27) due to triglyceride values > 4.2 mmol/L; ^†^: C-Reactive Protein data were excluded for two participants (HP *n* = 30) because of isolated values > 10 mg/L; ^#^: single medication combining two classes of oral hypoglycaemic agents. Abbreviations: BMI: body mass index; DPP-4: Dipeptidyl Peptidase-4; GLP-1: glucagon-like peptide 1; HbA1c: glycosylated haemoglobin; HDL: high-density lipoprotein; HOMA2-IR: homeostasis model assessment of insulin resistance; HC, higher-carbohydrate diet; HP, higher-protein diet; km/h, kilometers per hour; LDL, low-density lipoprotein; T2DM, type 2 diabetes mellitus.

**Table 2:** Energy, macronutrient composition, and physical activity results following weight loss (week 12) and weight maintenance (week 24) phases by dietary patterns.

	Phase 1: Weight Loss			Phase 2: Weight Maintenance		
	HP (*n* = 31)	HC (*n* = 28)	Diet *	HP (*n* = 23)	HC (*n* = 22)	Diet *
Energy (kJ)	6236 ± 617	5945 ± 866	0.14	7267 ± 1022	6975 ± 1038	0.35
Carbohydrate (g)	132.4 ± 18.6	179.6 ± 22.2	<0.001	149.2 ± 18.8	199.3 ± 23.6	<0.001
Carbohydrate (% en)	34.5 ± 3.7	49.6 ± 3.9	<0.001	33.6 ± 3.2	47.2 ± 4.5	<0.001
Protein (g)	107.1 ± 10.0	73.8 ± 10.0	<0.001	121.3 ± 19.6	82.1 ± 12.5	<0.001
Protein (% en)	29.4 ± 2.3	21.3 ± 1.7	<0.001	28.5 ± 2.8	20.1 ± 1.5	<0.001
Total Fat (g)	50.9 ± 6.9	36.1 ± 9.7	<0.001	62.2 ± 10.4	47.8 ± 11.7	<0.001
Total Fat (% en)	30.1 ± 2.4	22.1 ± 3.8	<0.001	31.6 ± 2.9	25.1 ± 3.6	<0.001
Saturated Fat (% en)	9.5 ± 1.1	6.4 ± 1.6	<0.001	10.3 ± 1.3	7.5 ± 1.5	<0.001
Saturated Fat (% TF)	34.8 ± 3.4	32.7 ± 4.6	0.04	36.0 ± 4.4	33.3 ± 3.9	0.04
MUFA (% TF)	46.7 ± 2.8	45.3 ± 4.3	0.14	46.3 ± 2.5	45.5 ± 3.8	0.37
PUFA (% TF)	18.5 ± 2.0	22.0 ± 5.1	0.001	17.7 ± 3.1	21.2 ± 4.2	0.002
Dietary Fiber (g)	25.1 ± 4.6	28.5 ± 4.3	0.005	25.7 ± 5.3	28.9 ± 5.0	0.04
Physical Activity						
Total Minutes/Week ^^^	211 ± 79	271 ± 133	0.08	194 ± 111	242 ± 158	0.24

Data are means ± SEM. *: Comparison of differences between diets analyzed with independent student *t*-tests. ^^^: Data from completers only (weight loss: HP *n* = 23, HC *n* = 22, weight maintenance: HP *n* = 23, HC *n* = 21). Abbreviations: % en: percent of energy; MUFA, monounsaturated fat; PUFA, polyunsaturated fat; % TF, percent of total fat; HP: higher-protein diet; HC: higher-carbohydrate diet.

**Table 3:** Changes in outcomes for weight, glycaemic control, body composition, blood pressure, HOMA2-IR, and CRP following 12 weeks weight loss (Week 12) and 12 weeks weight maintenance (Week 24).

						*p* Value		
	Week 12	Change in Phase 1 *	Week 24	Change in Phase 2 ^^^	Overall Change ^§^	Diet	Time	Group × Time
Weight (kg)						0.20	<0.001	0.47
HP	89.3 ± 2.7	−8.0 ± 0.80	88.4 ± 2.8	−1.0 ± 0.6	−8.9 ± 1.3			
HC	94.0 ± 2.9	−7.6 ± 0.8	93.8 ± 2.9	−0.2 ± 0.6	−7.7 ± 1.3			
Body Mass Index (BMI, kg/m^2^)						0.65	<0.001	0.53
HP	31.5 ± 0.85	−2.80 ± 0.25	31.2 ± 0.91	−0.30 ± 0.19	−3.10 ± 0.39			
HC	31.9 ± 0.89	−2.56 ± 0.26	31.9 ± 0.99	−0.01 ± 0.20	−2.57 ± 0.40			
HbA1c (%)						0.78	<0.001	0.19
HP	6.5 ± 0.2	−1.53 ± 0.20	6.7 ± 0.2	0.19 ± 0.16	−1.34 ± 0.21			
HC	6.8 ± 0.2	−1.30 ± 0.20	6.6 ± 0.2	−0.20 ± 0.16	−1.50 ± 0.22			
Fasting Glucose (mmol/L)						0.66	<0.001	0.74
HP	6.8 ± 0.3	−2.3 ± 0.5	7.0 ± 0.4	0.24 ± 0.3	−2.1 ± 0.6			
HC	6.5 ± 0.3	−2.8 ± 0.6	6.8 ± 0.5	0.30 ± 0.3	−2.5 ± 0.6			
Fasting Insulin (mU/L)						0.50	<0.001	0.52
HP	13.5 ± 1.4	−8.1 ± 1.8	12.1 ± 1.5	−1.4 ± 1.2	−9.6 ± 2.1			
HC	13.9 ± 1.4	−8.9 ± 1.9	14.9 ± 1.6	0.9 ± 1.3	−7.9 ± 2.2			
Medication Effect Score						0.43	0.02	0.08
HP	0.90 ± 0.16	−0.21 ± 0.06	0.97 ± 0.15	0.07 ± 0.04	−0.13 ± 0.06			
HC	1.18 ± 0.16	−0.04 ± 0.06	1.13 ± 0.16	−0.05 ± 0.04	−0.09 ± 0.06			
Waist circumference (cm)						0.75	<0.001	0.47
HP	104.2 ± 2.1	−8.5 ± 0.9	103.2 ± 2.2	−1.0 ± 0.6	−9.6 ± 1.2			
HC	105.6 ± 2.2	−7.0 ± 0.9	105.0 ± 2.3	−0.6 ± 0.6	−7.5 ± 1.2			
Fat mass (kg) ^†^						0.25	<0.001	0.44
HP	31.8 ± 1.9	−6.7 ± 0.7	30.7 ± 2.0	−1.1 ± 0.5	−7.8 ± 1.0			
HC	35.1 ± 2.0	−5.5 ± 0.7	34.7 ± 2.0	−0.4 ± 0.5	−5.9 ± 1.1			
Fat-Free Mass (kg) ^†^						0.63	<0.001	0.56
HP	57.6 ± 2.1	−1.2 ± 0.4	57.8 ± 2.0	0.10 ± 0.3	−1.1 ± 0.5			
HC	58.9 ± 2.2	−1.8 ± 0.4	59.0 ± 2.1	0.13 ± 0.3	−1.7 ± 0.5			
SBP (mmHg) ^#^						0.25	<0.001	0.07
HP	123.9 ± 2.4	−7.9 ± 1.9	119.5 ± 2.3	−4.4 ± 1.8	−12.3 ± 1.8			
HC	123.8 ± 2.5	−11.2 ± 2.0	125.2 ± 2.3	1.4 ± 1.8	−9.8 ± 1.9			
DBP (mmHg) ^#^						0.51	<0.001	0.07
HP	72.3 ± 1.4	−6.2 ± 1.2	70.7 ± 1.6	−1.5 ± 1.1	−7.7 ± 1.4			
HC	71.9 ± 1.4	−7.2 ± 1.2	74.1 ± 1.6	2.3 ± 1.1	−4.9 ± 1.4			
HOMA2_IR ^ǂ^						0.94	<0.001	0.45
HP	1.82 ± 0.20	−1.21 ± 0.25	1.60 ± 0.21	−0.22 ± 0.19	−1.44 ± 0.32			
HC	1.64 ± 0.21	−1.19 ± 0.25	1.79 ± 0.21	0.14 ± 0.19	−1.04 ± 0.33			
CRP (mg/L) ^¥^						0.28	<0.001	0.99
HP	2.52 ± 0.60	−1.19 ± 0.36	2.37 ± 0.53	−0.15 ± 0.37	−1.33 ± 0.37			
HC	3.63 ± 0.62	−0.94 ± 0.34	2.85 ± 0.55	−0.78 ± 0.37	−1.72 ± 0.37			
Treadmill Speed (km/h)								
HP	5.3 ± 0.2	0.67 ± 0.12	5.6 ± 0.2	0.35 ± 0.12	1.02 ± 0.18	0.99	<0.001	0.70
HC	5.6 ± 0.2	0.53 ± 0.13	5.5 ± 0.2	0.28 ± 0.13	0.8 ± 0.19			

Data is represented by means ± SEM for the 61 participants who commenced the study. HP: higher-protein diet (*n* = 32); HC: higher-carbohydrate diet (*n* = 29). *: Change from weight loss (Weeks 0–12); ^^^: Change from weight maintenance (Weeks 12–24); ^§^: Overall change (Weeks 0-24). ^#^ blood pressure data for one participant at Week 24 excluded from analysis as an extreme outlier (HP *n* = 31); ^ǂ^: HOMA2-IR could not be calculated on 12 participants due to insulin therapy and one participant due to high fasting insulin level at baseline (HP *n* = 25, HC *n* = 23), ^¥^: Isolated CRP values > 10 mg/L were not included for six participants (HP *n* = 27, HC *n* = 28). Abbreviations: CRP, C-reactive protein; HOMA2-IR, homeostasis model of assessment index 2 of insulin resistance; km/h, kilometers per hour; SBP, systolic blood pressure; DBP, diastolic blood pressure.

**Table 4:** Changes in outcomes for blood lipids following 12 weeks weight loss (Week 12) and 12 weeks weight maintenance (Week 24).

						*p* Value		
	Week 12	Change *	Week 24	Change ^^^	Overall Change	Diet	Time	Group × Time
Total cholesterol (mmol/L)						0.93	<0.001	0.049
HP	4.1 ± 0.2	−0.6 ± 0.1	4.3 ± 0.2	0.3 ± 0.1	−0.4 ± 0.1			
HC	4.2 ± 0.2	−0.2 ± 0.1	4.4 ± 0.2	0.2 ± 0.1	−0.03 ± 0.1			
LDL-C (mmol/L) ^§^						0.94	0.02	0.18
HP	2.3 ± 0.2	−0.3 ± 0.1	2.4 ± 0.2	0.02 ± 0.1	−0.3 ± 0.1			
HC	2.4 ± 0.2	−0.1 ± 0.1	2.5 ± 0.2	0.1 ± 0.1	−0.004 ± 0.1			
HDL-C (mmol/L)						0.87	0.004	0.14
HP	1.1 ± 0.05	−0.05 ± 0.03	1.2 ± 0.05	0.1 ± 0.04	0.03 ± 0.03			
HC	1.2 ± 0.05	0.02 ± 0.03	1.3 ± 0.06	0.1 0.04	0.1 ± 0.03			
Triglycerides (mmol/L)						0.43	<0.001	0.15
HP	1.3 ± 0.1	−0.8 ± 0.2	1.6 ± 0.2	0.3 ± 0.2	−0.4 ± 0.2			
HC	1.5 ± 0.1	−0.5 ± 0.2	1.5 ± 0.2	−0.03 ± 0.2	−0.6 ± 0.2			

Data is represented by means ± SEM for the 61 participants who commenced the study. HP: higher-protein diet (*n* = 32); HC: higher-carbohydrate diet (*n* = 29). *: Change from baseline, ^^^ Change from phase 1; ^§^: LDL cholesterol could not be calculated for five participants at baseline (HP *n* = 29, HC *n* = 27) due to triglycerides levels > 4.2 mmol/L. Abbreviations: HDL-C, high-density lipoproteins; LDL-C, low-density lipoprotein cholesterol.

## Abbreviations

The following abbreviations are used in this manuscript:

ADA	American Diabetes Association
ARENA	Alliance for Research in Exercise, Nutrition and Activity
BGL	blood glucose level
BMI	body mass index
BP	blood pressure
CRP	C-reactive protein
CSIRO	Commonwealth Scientific and Industrial Research Organization
CVD	cardiovascular disease
DBP	diastolic blood pressure
DEXA	dual energy x-ray absorptiometry
DPP-4	Dipeptidyl Peptidase-4
GLP-1	glucagon-like peptide 1
HbA1c	glycosylated haemoglobin
HC	higher-carbohydrate, lower-protein
HDL	high-density lipoproteins
HOMA2-IR	homeostasis model of assessment index 2 of insulin resistance
HP	higher-protein, lower-carbohydrate
ITT	intention-to-treat
kg	kilogram
kJ	kilojoule
LDL	low-density lipoprotein
MES	medication effect score
MUFA	monounsaturated fat
PUFA	polyunsaturated fat
RCT	randomised controlled trial
RPE	rate of perceived exertion
SBP	systolic blood pressure
T2DM	type 2 diabetes mellitus
% en	percent of energy
% TF	percent of total fat
