# Correction: Watson, N.; et al. Effects of Low-Fat Diets Differing in Protein and Carbohydrate Content on Cardiometabolic Risk Factors during Weight Loss and Weight Maintenance in Obese Adults with Type 2 Diabetes. *Nutrients* 2016, *8*, 289

**DOI:** 10.3390/nu9121283

**Published:** 2017-11-24

**Authors:** Nerylee Watson, Kathryn Dyer, Jonathan Buckley, Grant Brinkworth, Alison Coates, Gaynor Parfitt, Peter Howe, Manny Noakes, Karen Murphy

**Affiliations:** 1 Alliance for Research in Exercise, Nutrition and Activity, Sansom Institute for Health Research, University of South Australia, GPO Box 2471, Adelaide, SA 5001, Australia; nerylee.watson@mymail.unisa.edu.au (N.W.); Kate.Dyer@unisa.edu.au (K.D.); Jon.Buckley@unisa.edu.au (J.B.); Alison.Coates@unisa.edu.au (A.C.); Gaynor.Parfitt@unisa.edu.au (G.P.); 2 Food and Nutrition, Commonwealth Scientific and Industrial Research Organization, P.O. Box 10041, Adelaide, SA 5000, Australia; grant.brinkworth@csiro.au (G.B.); manny.noakes@csiro.au (M.N.); 3 Clinical Nutrition Research Centre, School of Biomedical Sciences and Pharmacy, University of Newcastle, University Drive, Callaghan, NSW 2308, Australia; Peter.Howe@newcastle.edu.au

The authors request the following corrections to their paper [1]. 

In the abstract, ‘HP diet (38% carbohydrate, 30% protein, 29% fat) to an isocaloric higher-carbohydrate diet (HC: 53%:21%:23%)’ was replaced with ‘HP diet (mean across both phases: 29% protein, 34% carbohydrate, 31% fat) to an isocaloric higher-carbohydrate diet (HC: 21%:48%:24%)’.

In Table 2, ‘Data are means ± SEM’ was replaced with ‘Data are means ± SD’.

The authors apologize for this oversight and any inconvenience caused to the readers by these changes, stating it does not affect the scientific results.
